# Nuclear pCHK1 as a potential biomarker of increased sensitivity to ATR inhibition

**DOI:** 10.1002/path.6032

**Published:** 2022-12-08

**Authors:** Vignesh Sundararajan, Tuan Zea Tan, Diana Lim, Yanfen Peng, Antje Margret Wengner, Natalie Yan Li Ngoi, Anand D Jeyasekharan, David Shao Peng Tan

**Affiliations:** ^1^ Cancer Science Institute of Singapore National University of Singapore Singapore Singapore; ^2^ Genomics and Data Analytics Core (GeDaC), Cancer Science Institute of Singapore National University of Singapore Singapore Singapore; ^3^ Department of Pathology National University Hospital Singapore Singapore; ^4^ Bayer AG, Pharmaceuticals Berlin Germany; ^5^ Department of Haematology‐Oncology National University Cancer Institute Singapore Singapore

**Keywords:** pCHK1, ATR inhibitor, elimusertib, BAY 1895344, biomarker, replicative stress response

## Abstract

Excessive genomic instability coupled with abnormalities in DNA repair pathways induces high levels of ‘replication stress’ when cancer cells propagate. Rather than hampering cancer cell proliferation, novel treatment strategies are turning their attention towards targeting cell cycle checkpoint kinases (such as ATR, CHK1, WEE1, and others) along the DNA damage response and replicative stress response pathways, thereby allowing unrepaired DNA damage to be carried forward towards mitotic catastrophe and apoptosis. The selective ATR kinase inhibitor elimusertib (BAY 1895344) has demonstrated preclinical and clinical monotherapy activity; however, reliable predictive biomarkers of treatment benefit are still lacking. In this study, using gene expression profiling of 24 cell lines from different cancer types and in a panel of ovarian cancer cell lines, we found that nuclear‐specific enrichment of checkpoint kinase 1 (CHK1) correlated with increased sensitivity to elimusertib. Using an advanced multispectral imaging system in subsequent cell line‐derived xenograft specimens, we showed a trend between nuclear phosphorylated CHK1 (pCHK1) staining and increased sensitivity to the ATR inhibitor elimusertib, indicating the potential value of pCHK1 expression as a predictive biomarker of ATR inhibitor sensitivity. © 2022 The Authors. *The Journal of Pathology* published by John Wiley & Sons Ltd on behalf of The Pathological Society of Great Britain and Ireland.

## Introduction

The integrity of human cellular DNA is a major obstacle to carcinogenesis and is constantly threatened by cellular stressors that compromise DNA replication [1]. These factors include exogenous agents such as ionizing radiation, ultraviolet radiation, hypoxia, chemical mutagens, and chemotherapeutic drugs, as well as endogenous stressors such as replication errors, reactive oxygen species, and shortage of nucleotide pools, which collectively induce so‐called ‘replication stress’. Under these conditions, replicative stress response (RSR) pathways are activated to allow time for DNA repair in order to maintain genomic integrity [2]. For instance, when the above‐mentioned stress agents lead to stalled replication forks and the generation of single‐stranded DNA, replication protein A (RPA) [3] is recruited onto the single‐stranded DNA. This further triggers the assembly of the RSR machinery components such as activated ataxia–telangiectasia and rad3‐related (ATR) kinase, topoisomerase 2‐binding protein 1 (TOPBP1), and DNA repair protein RAD17 [4]. ATR‐mediated phosphorylation of checkpoint kinase 1 (CHK1) induces cell cycle arrest by interrupting phase‐transition checkpoints, allowing the cells to resolve blocked fork sites, preventing collapse of stalled replication forks as well as averting rapid firing of late replicating origins [5, 6]. Therefore, inhibition of ATR and CHK1 could lead to the accumulation of replicative stress, eventually resulting in mitotic catastrophe and apoptosis [7].

Targeting the RSR pathway has emerged as a promising therapeutic approach in the context of recurrent/drug‐resistant tumors [8, 9, 10]. In particular, elimusertib (BAY 1895344; Bayer AG, Leverkusen, Germany) is a selective ATR kinase inhibitor that has demonstrated antitumor activity *in vitro*, *in vivo*, and in phase I clinical trials [11, 12, 13, 14]. At present, no reliable predictive biomarkers have been identified that select patients most likely to be susceptible to ATR inhibition. Although ATM (ataxia–telangiectasia mutated) loss is thought to confer reliance on ATR and has been highlighted as a potential biomarker for elimusertib sensitivity, its predictive value remains unconfirmed based on pre‐clinical and clinical studies to date. In this study, we subjected a panel of 24 cancer cell lines with varying sensitivity to elimusertib, to multiplexed gene expression profiling using a panel of 770 genes associated with canonical cancer pathways. Subsequent gene set enrichment analysis on this panel revealed an abundance of DNA damage response (DDR) or cell‐cycle checkpoint gene expression, including checkpoint kinase 1 (*CHK1*), which correlated with increased sensitivity to elimusertib. Using a panel of ovarian cancer cell lines, we show a significant correlation between increased nuclear phosphorylated checkpoint kinase 1 (pCHK1) staining and increased sensitivity to elimusertib. In addition, using a tissue microarray of cell line‐derived xenografts, we found a trend between increased nuclear pCHK1 staining and increased sensitivity to elimusertib. Furthermore, we observed the presence of nuclear pCHK1 expression in clinical pre‐treatment tumor samples of patients who had derived clinical benefit from elimusertib, denoting the use of pCHK1 as a potential biomarker for identifying patients susceptible to ATR inhibition.

## Materials and methods

### Cell lines and *in vitro* proliferation assays

The cell lines used in the study were grown as described previously [13] and were maintained in different media (supplementary material, Table S1). The anti‐proliferative activity of elimusertib was evaluated by plating the cells in their appropriate growth medium in 96‐well format, and by incubating them with increasing concentrations of elimusertib (3 nm–3 μm) for 4 days. We measured cell viability using either the CellTiter‐Glo® Cell Viability Assay (Promega, Madison, WI, USA; Cat # G7570) or Cell Counting Kit‐8 (CCK‐8; Dojindo Molecular Technologies, Inc., Kumamoto, Japan; Cat # CK04‐20), and luminescence/absorbance was recorded using a plate reader (VICTOR V, PerkinElmer, Waltham, MA, USA). We performed all measurements in quadruplicates and normalized with respect to the cell number at the beginning of the treatment and to the cell number of the untreated control group. The half‐maximal inhibitory concentration (IC_50_) was determined using a four‐parameter logistic regression model. Methods for ‘siRNA treatment and western blotting’ are elaborated in Supplementary materials and methods.

### Generation of cell line‐derived xenograft models

Cancer cells from mid‐log phase (70% confluent) cultures were subcutaneously inoculated by injecting 100 μl of cell suspension into the flanks of female SCID beige mice (5–6 weeks, 20–22 g; Envigo, Indianapolis, IN, USA). When the tumors reached a predetermined size of 25–50 mm^2^, mice were randomized into treatment and control groups (*n* = 10–11 mice per group), and treated with elimusertib. Elimusertib was formulated in PEG400/ethanol/water (60:10:30) and per‐oral treatment was conducted in 3 days on/4 days off. Tumor response was assessed by measuring the tumor area (length × width) using a caliper. Animal body weight was monitored as a measure of treatment‐related toxicity. Tumor area and body weight were determined two or three times per week. Changes in body weight were considered as a measure of treatment‐related toxicity (>10% loss of body weight was considered critical and treatment dosing was held until recovery; >20% loss of body weight was considered toxic and treatment was terminated). Mice were euthanized when showing signs of toxicity (>20% body weight loss), or when tumors reached a maximum area of 225 mm^2^. Tumor growth inhibition is presented as T/C (treatment/control) ratio, calculated with tumor areas at the end of the study. Relative tumor growth inhibition based on tumor area T/C ratio was calculated using the following formula: T/C ratio = {[(tumor area of treatment group at day *x*) – (tumor area of treatment group before treatment)]/[(tumor area of control group at day *x*) – (tumor area of control group before treatment)]}. T/C ratio <0 to <0.3 was considered a good response; T/C ratio >0.3 to <0.6 was considered a moderate response; and T/C ratio >0.6 to 0.8 was considered a weak response (no biological relevance). T/C ratio of 0.9–1.0 meant that the treatment was considered to be inactive (no biological relevance).

### Multiplex immunohistochemistry staining

A tissue microarray (TMA) slide with core sections from 15 xenograft tissues (thickness = 3 μm) was constructed on a poly‐l‐lysine‐coated microscope glass slide. Multiplex immunofluorescence (IF) staining was performed on cell and TMA slides using the Opal™ Tyramide Signal Amplification (TSA) system following the manufacturer's protocol. Heat‐induced epitope retrieval (HIER) was conducted on the slide, followed by incubation with the primary antibody for 60 min at room temperature. The slide was then washed with TBST buffer for 5 min and then incubated with HRP‐labeled secondary antibody at room temperature for 5 min. Subsequently, a tyramide‐based fluorescent reagent (a selection of Opal fluorophores, listed in supplementary material, Table S2) was applied. The staining efficiency of each primary antibody was checked using a fluorescence microscope. The slide was subsequently baked in a microwave oven to inactivate HRP in preparation for the next antibody application. After all antibodies had been stained, nuclear counterstain (DAPI; Sigma, St Louis, MO, USA; Cat # D9542) was added for 10 min and the slide was washed twice in TBST for 5 min each. The slide was mounted using an appropriate mounting medium. A total of four primary antibodies were used for staining: geminin (Protein Tech, Rosemont, IL, USA; Cat #10802‐1‐AP; 1:100); phospho‐ATR (Ser428; Abcam, Cambridge, UK; Cat # ab178407; 1:100); phospho‐CHK1 (Ser345; Cell Signaling Technology, Danvers, MA, USA; Cat # 2348; 1:100); and γH2AX (Millipore, Burlington, MA, USA; Cat # 05636; 1:100). The performance of the markers was tested using control slides prepared from RAJI cells with/without treatment with 50 μm etoposide (Sigma; Cat # 1268808) for 24 h which had been embedded in paraffin blocks after treatment. A detailed description of ‘single marker immunohistochemistry’ and ‘multispectral imaging and signal quantification’ is provided in Supplementary materials and methods.

### NanoString gene expression profiling and processing

Total RNA was extracted from cell lines using a QIAGEN RNeasy Mini Kit (QIAGEN, Hilden, Germany; Cat #74104) following the manufacturer's protocol. An Agilent 2100 Bioanalyzer (Agilent, Santa Clara, CA, USA) was used to check the quality and quantity of extracted RNA from samples. Only RNA with length greater than 300 nucleotides (i.e. functional RNA) was considered for quantity calculations for downstream use. Aliquots (100 ng) of unamplified total RNA were hybridized with the NanoString PanCancer Pathway CodeSet and Capture ProbeSet (NanoString Technologies Inc., Seattle, WA, USA) at 65 °C for at least 16 h but not more than 48 h in a thermal cycler. The hybridized RNA samples were then loaded onto the NanoString nCounter system for gene expression analysis. Gene expression normalization of NanoString data was performed using nSolver analysis software version 3.0 (NanoString Technologies Inc.). The raw counts from NanoString were subjected to background subtraction, positive control normalization, and reference gene normalization. The normalized counts were then log2‐transformed prior to downstream analysis.

### Human tumor tissue samples and clinical data

Ethics approval for these procedures was obtained from the National Health Group Domain Specific Review Board (DSRB), Reference: 2013/00705. Human tumor tissue samples were obtained with patient consent from the National University Hospital Singapore (NUHS) Tissue Repository. Patient clinical data including best tumor response by investigator‐evaluated Response Evaluation Criteria in Solid Tumours (RECIST) version 1.1 and progression‐free survival (PFS) were retrospectively retrieved from medical records.

### GDSC drug sensitivity analysis

The drug sensitivity of cancer drugs was extracted from Genomics of Drug Sensitivity in Cancer (GDSC2) release 8.2 [15]. pCHK1 levels were extracted from MD Anderson Cell Lines Project (https://tcpaportal.org/mclp/#/) version 1.1.

## Results

### Increased expression of DNA damage response (DDR) pathway genes is associated with sensitivity to ATR inhibition

**Figure 1:**
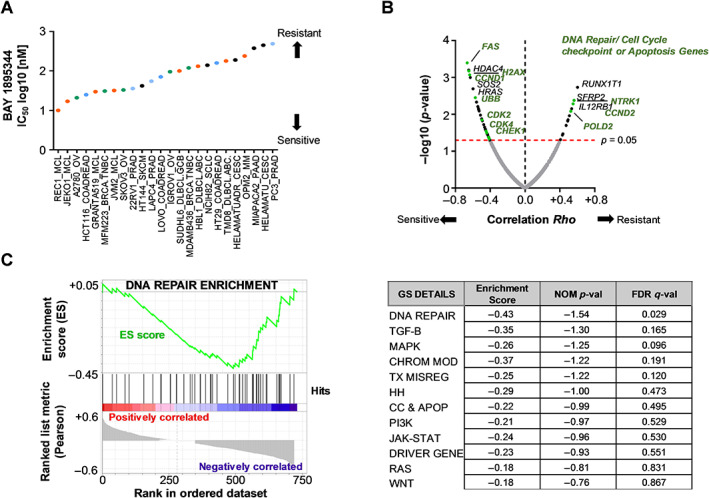
Members of the DNA repair pathway as potential indicators of sensitivity to treatment with elimusertib. (A) Dot plot showing IC_50_ values for elimusertib in a panel of human cancer cell lines from multiple cancer entities. MCL, mantle cell lymphoma; OV, ovarian carcinoma; COADREAD, colorectal carcinoma; BRCA.TNBC, *BRCA*‐mutated triple‐negative breast cancer; PRAD, prostate carcinoma; SKCM, skin cutaneous melanoma; DLBCL, diffuse large B‐cell lymphoma; GCB, germinal center B‐cell subtype; ABC, activated B‐cell lymphoma; SCLC, small cell lung cancer; CESC, cervical carcinoma; MM, multiple myeloma; PAAD, pancreatic carcinoma. (B) Volcano plot showing correlation (*Rho*) of IC_50_ with differentially expressed genes (mRNA expression) in the PanCancer pathway panel of the NanoString nCounter system. Genes associated with DNA repair/cell cycle checkpoint or apoptosis are denoted in green. (C) Left: gene set enrichment analysis (GSEA) plot showing that the DNA repair gene set (FDR < 0.05) is enriched in sensitive cell lines. Right: list of enriched gene sets showing that the DNA repair pathway is the only significant hit (FDR *q*‐value = 0.029).

Using a panel of 24 cancer cell lines from multiple cancer types, we assayed for anti‐proliferative activity of elimusertib and identified a spectrum of cell lines with varying sensitivity, with IC_50_ values ranging from approximately 10 to 500 nm (Figure 1A). Of these 24 cell lines, the mantle cell lymphoma cell lines showed the greatest sensitivity with the lowest IC_50_ values (Figure 1A). To identify an underlying gene expression signature that distinguishes elimusertib‐resistant from elimusertib‐sensitive cancers, we performed gene expression profiling using the NanoString PanCancer pathway panel of 770 genes. We performed correlation analysis between the expression levels of 770 genes associated with canonical cancer pathways and *in vitro* elimusertib IC_50_ using the Spearman correlation coefficient rank test. Fifty‐seven significantly correlated genes were identified (supplementary material, Table S3). In particular, DDR targets including DNA repair/cell‐cycle checkpoint or apoptosis genes were significantly correlated with elimusertib response *in vitro* (Figure 1B). Among these genes, higher expression levels of *FAS*, *H2AX*, *CCND1*, *UBB*, *CDK2*/*4*, and *CHEK1* correlated with lower IC_50_ of elimusertib, suggesting greater sensitivity. In particular, *CHEK1* codes for the protein kinase Chk1, which is phosphorylated by ATR following DNA damage induced by a variety of agents, and has an essential role in transducing a delay signal to the cell cycle machinery in the presence of DNA damage. Furthermore, gene set enrichment analysis (GSEA) of candidate genes correlating with higher sensitivity to elimusertib indicated that they were significantly enriched with the DNA repair components (*H2AFX*, *FEN1*, *CHEK1*, *XRCC4*, *BRIP1*, *FANCC*, *RAD51*, *BRCA1*, *BRCA2*, *PCNA*, *FANCA*, *FANCB*) of the DDR pathway (Figure 1C). These results indicate that higher expression of members of the DDR pathway could be a potential indicator of increased sensitivity to ATR inhibition using elimusertib.

### Phosphorylated CHK1 correlates with ATR inhibition sensitivity *in vitro* and *in vivo*

**Figure 2:**
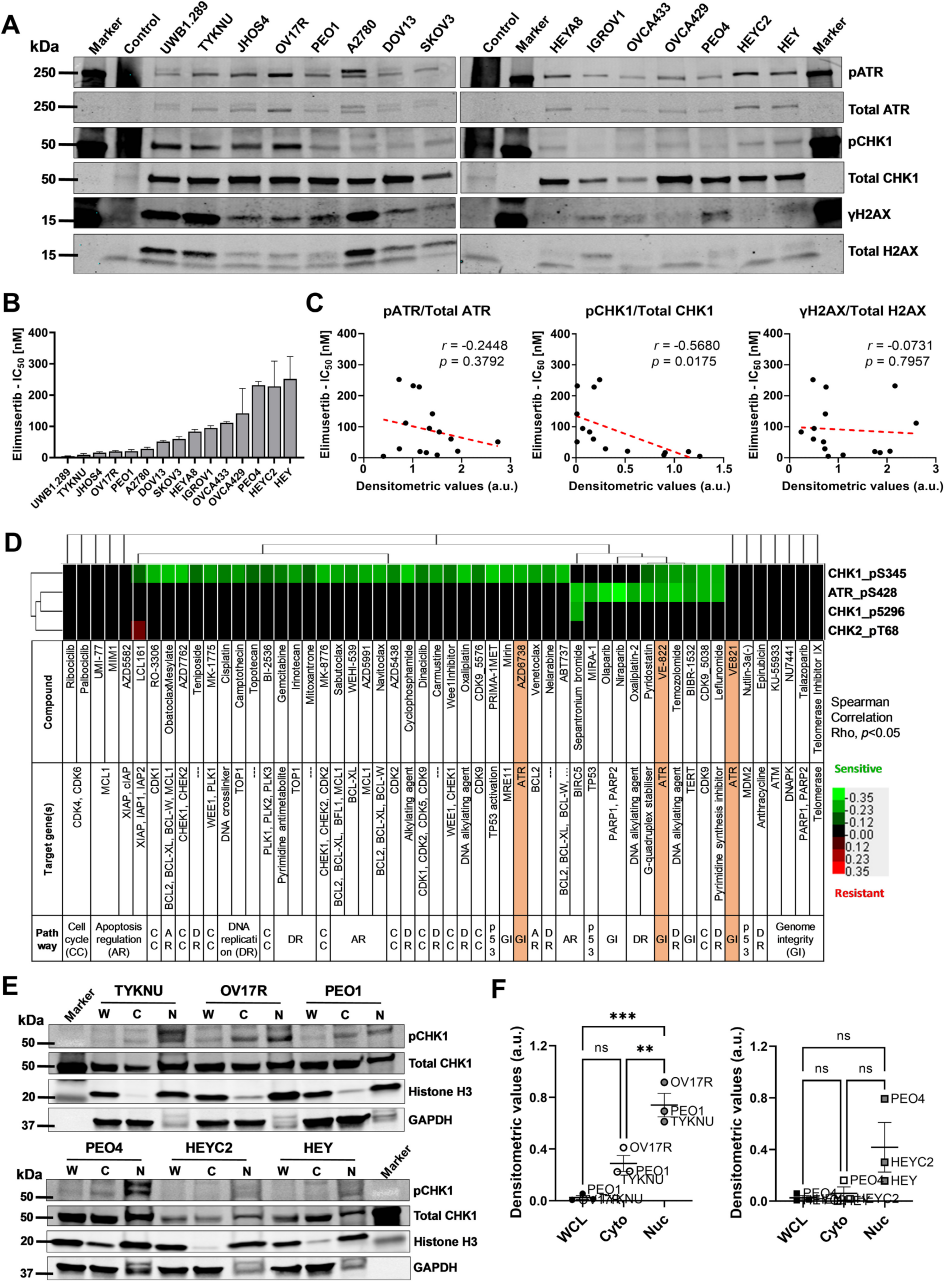
pCHK1 staining correlates with elimusertib sensitivity *in vitro*. (A) Western blotting for pATR, total ATR, pCHK1, total CHK1, γH2AX, and total H2AX in the ovarian cancer cell line panel. SKOV3 cells treated with 8 mm hydroxyurea for 4 h was used a control sample. (B) Bar chart showing elimusertib half‐maximal inhibitory concentration (IC_50_) of the ovarian cancer cell line panel. Data presented are from three biological replicates with SEM error bar. (C) Dot plots showing correlations between elimusertib IC_50_ (*y*‐axes) and densitometric values [*x*‐axes, arbitrary units (a.u.)] of activated protein expression (phospho‐protein normalized with respective total protein) of ATR (left), CHK1 (middle), and H2AX (right). Pearson correlation coefficients (*r*) and *P* values (*p*) are denoted. (D) Heatmap showing that IC_50_ (log10) of compounds targeting DNA damage or replication stress protein expression correlated with activated ATR and CHK1/2. ATR inhibitors are highlighted in orange. (E) Western blots for pCHK1 and total CHK1 in whole cell lysate (W), cytoplasmic (C), and nuclear (N) fractions of elimusertib‐sensitive (upper panel: TYKNU, OV17R, PEO1) and elimusertib‐resistant (lower panel: PEO4, HEYC2, HEY) cell lines. Nuclear marker Histone H3 and cytoplasmic marker GAPDH served as respective cell fractionation controls and revealed minimal cross‐contamination. (F) Dot plots showing densitometric values [*y*‐axes, arbitrary units (a.u.)] of activated CHK1 levels (pCHK1 normalized with total CHK1) in the elimusertib‐sensitive (left) and elimusertib‐resistant (right) ovarian cancer cell lines. Mean ± SEM from three cell lines were plotted. One‐way ANOVA and Tukey's multiple comparison tests were used; **p < 0.01 and ***p < 0.001. ns, not significant.

In view of the observed correlation between *CHEK1* gene expression and increased sensitivity to elimusertib, we subsequently assessed whether activation of proteins of the ATR–CHK1 axis [phosphorylated ATR (pATR), phosphorylated CHK1 (pCHK1), and gamma‐H2AX (γH2AX)] would be a potential biomarker of elimusertib sensitivity. Phosphorylated ATR‐mediated activation of its downstream targets, such as γH2AX and CHK1, was also assessed as a direct indicator of cellular response to DNA damage occurring at several phases of the cell cycle [16, 17, 18]. Accordingly, we assessed the endogenous expression levels of the above‐mentioned DDR proteins (phosphorylated and total levels) in a panel of 15 ovarian cancer cell lines (Figure 2A). The cell viability assay revealed a spectrum of elimusertib sensitivity amongst this panel of cell lines (Figure 2B). Subsequent correlation of the expression levels of activated basal proteins with IC_50_ of elimusertib identified a significant correlation between endogenous activated CHK1 levels and elimusertib sensitivity (*rho* = −0.5680; *p* = 0.0175), denoting that cell lines with a lower IC_50_ of elimusertib have higher endogenous pCHK1 expression (Figure 2C). However, IC_50_ of elimusertib showed no correlation with γH2AX (*rho* = −0.0731; *p* = 0.7957) and a non‐significant correlation with pATR protein levels (*rho* = −0.2448; *p* = 0.3792), indicating that endogenous expression of these two proteins is not associated with increased elimusertib sensitivity (Figure 2C). Along this line, we extracted protein expression data from a data repository, GDSC2 [15], and correlated drug dose response (IC_50_ values) to other DDR and RSR inhibitors (Figure 2D). Similar to what we have demonstrated, among the DDR pathway proteins that were analyzed, the protein levels of pCHK1 (pCHK1_Ser345) correlated negatively with the IC_50_ of compounds targeting the DDR pathway, including multiple ATR inhibitors such as VE‐821, VE‐822, and AZD6738 (Figure 2D). To further characterize whether localization of pCHK1 could be associated with drug sensitivity, we measured pCHK1 and total CHK1 levels in whole cell, cytoplasmic, and nuclear fractions, and identified a significant enrichment of pCHK1 expression in the nuclear fractions of elimusertib‐sensitive cell lines (TYKNU, OV17R, and PEO1, Figure 2E, upper panel; Figure 2F, left plot) in comparison to the elimusertib‐resistant cell lines (PEO4, HEYC2, and HEY, Figure 2E, lower panel; Figure 2F, right plot). These results suggest that enrichment of nuclear activated CHK1 as indicated by pCHK1 levels (a critical substrate of ATR) is a potential candidate for predicting sensitivity to compounds targeting the DDR pathway, especially to ATR inhibitors.

**Figure 3:**
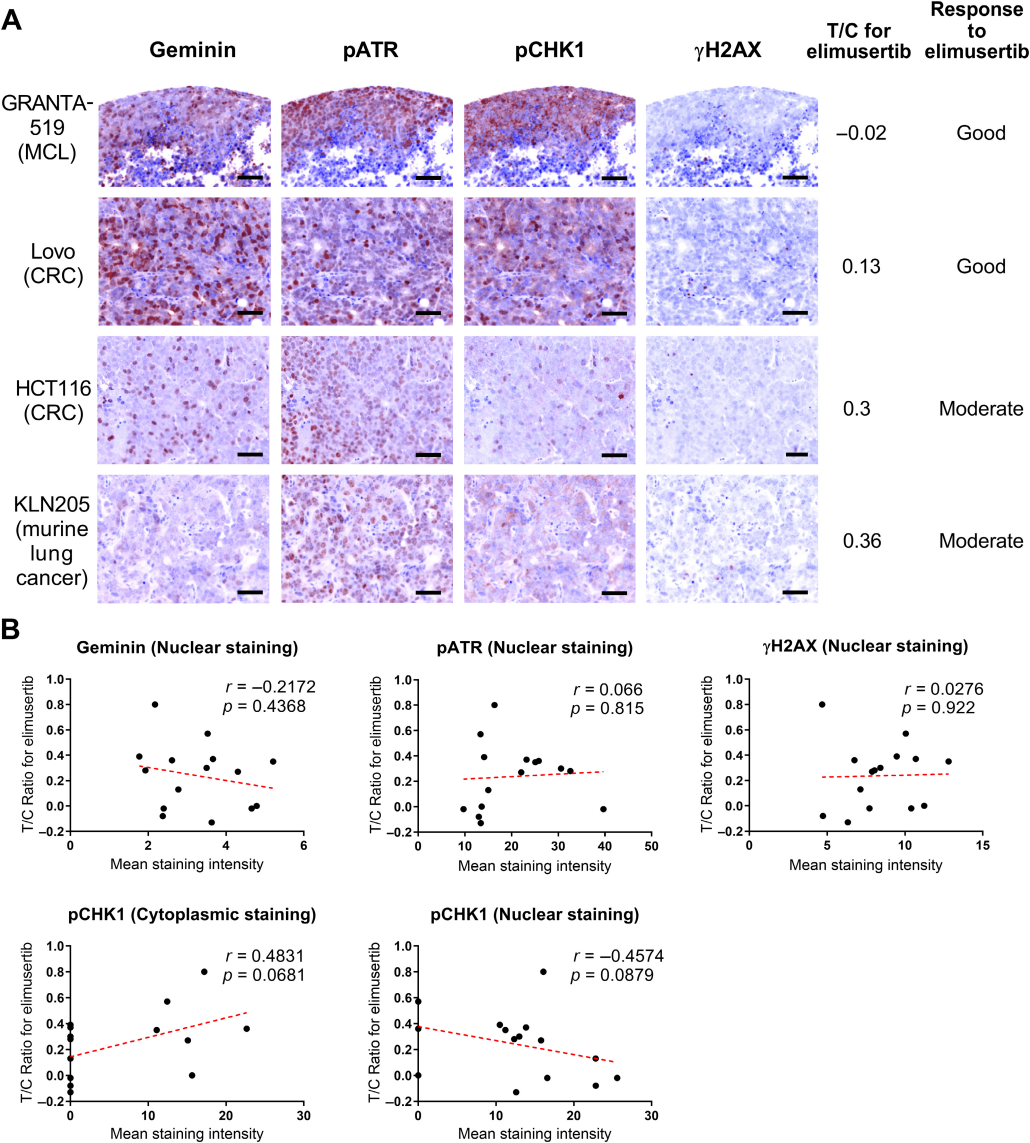
Nuclear pCHK1 staining correlates with elimusertib sensitivity *in vivo*. (A) Representative baseline staining for geminin, pATR, pCHK1, and γH2AX in tissue samples from cell line‐derived xenograft tumors of GRANTA‐519 (MCL, mantle cell lymphoma), Lovo and HCT116 (CRC, colorectal carcinoma), and KLN205 prior to elimusertib treatment. Scale bars: 50 μm. The treatment/control (T/C) ratio denoting good (<0.3) and moderate (0.3–0.6) response to elimusertib is also shown. Each image was obtained through multiplex immunofluorescence staining and is presented as pseudo‐color, mimicking a DAB‐based IHC stain for ease of visualization. (B) Dot plots showing that the T/C ratio for elimusertib correlated to mean staining intensities for geminin, pATR, γH2AX, and pCHK1 (cytoplasmic and nuclear) derived from xenografts. Pearson correlation coefficients (*r*) and *P* values (*p*) are given.

We extended our analysis of DDR markers predicting elimusertib sensitivity to *in vivo* models. Subsequently, we generated 15 cell line‐derived xenografts and assessed basal protein expression to identify potential biomarkers predicting sensitivity to elimusertib. A list of the tissue samples of cell line‐derived xenografts and the respective details of elimusertib responses of xenograft tumors, measured as treatment/control (T/C) ratios, are provided in Table 1. Tissue microarray (TMA) slides were constructed from xenograft tissues and stained for DDR markers. To identify proliferative cancer cells, we stained for geminin, which is a marker selectively correlated with the proliferative phases of the cell cycle and is regarded as a predictor of worse clinical outcome in high‐grade tumors [19, 20, 21]. The stained slides were subjected to multimodal, automated imaging using the Vectra 3 system (PerkinElmer Inc., Waltham, MA, USA), which allows for unmixing of more than five different fluorophores per sample, along with a machine learning‐based image‐processing algorithm for quantitation. The average mean intensities of stained protein markers for each of the xenograft tumor samples were calculated (Table 1). Overall, varying mean intensities were observed across different xenograft samples for each of the markers tested. In particular, xenografts derived from cell lines that showed a good response (response rating < 0.3) to elimusertib, such as GRANTA‐519 (MCL) and Lovo (CRC), also showed strong nuclear geminin, pATR, and pCHK1 signals, whereas those that showed a moderate response (response rating 0.3–0.6) to elimusertib, such as HCT116 (CRC) and KLN205 (murine lung cancer), showed moderate signals (Figure 3A). Furthermore, for each of the stained markers, the mean signal intensities on the xenograft tissue samples were correlated with the respective xenograft responses to elimusertib (measured by the T/C ratios of tumor area at cull). The nuclear signal intensities of geminin, pATR, and γH2AX did not show any correlation with elimusertib response (Figure 3B, upper panel). However, staining of pCHK1 showed a potential correlation with elimusertib sensitivity – higher cytoplasmic pCHK1 levels showed a trend towards lower elimusertib sensitivity (Figure 3B, lower panel plots, *r* = 0.4831, *p* = 0.06) and, in contrast, higher nuclear pCHK1 levels showed a trend towards higher elimusertib sensitivity (Figure 3B, lower panel plots, *r* = −0.4574, *p* = 0.0879). These observed trends indicate that the specific enrichment of pCHK1 levels in the nucleus might be regarded as an indicator of higher elimusertib sensitivity in xenograft tumors.

**Table 1:** Cell line‐derived xenografts, respective elimusertib responses based on treatment/control (T/C) ratios, and mean intensities of stained markers in the xenograft tissue samples.

S. No.	Cell line‐derived xenograft tissue sample	Cell line type	Response to elimusertib		Geminin nuclear mean intensity	pATR nuclear mean intensity	pCHK1 nuclear mean intensity	pCHK1 cytoplasmic mean intensity	γH2AX nuclear mean intensity
			T/C for elimusertib	Rating of response*					
1	22Rv1	Prostate	0.35	Moderate	5.212	25.088	11.209	11.09	12.808
2	A549	NSCLC	0.28	Good	1.93	32.587	12.343	0	8.048
3	CT26	Murine CRC	0.57	Moderate	3.527	13.343	0	12.448	10.058
4	GRANTA‐519	MCL	−0.02	Good	4.657	39.675	25.574	0	10.404
5	HCT116	CRC	0.3	Moderate	3.502	30.595	12.985	0	8.421
6	HeLa‐MaTu‐ADR	Cervix	0.37	Moderate	3.665	23.198	13.858	0	10.697
7	HT‐29	CRC	0.39	Moderate	1.772	14.131	10.486	0	9.471
8	KLN205	Murine lung	0.36	Moderate	2.613	25.825	0	22.665	6.757
9	LapC4	Prostate	0.27	Good	4.303	22.056	15.797	15.098	7.892
10	LNCap	Prostate	0	Good	4.788	13.587	0	15.644	11.241
11	Lovo	CRC	0.13	Good	2.776	15.01	22.81	0	7.141
12	MDA‐MB‐231	Breast	0.2	Good	2.552	15.984	15.076	0	5.052
13	MFM223	TNBC	0.8	Weak	2.174	16.344	16.083	17.213	4.679
14	NCI‐H82	SCLC	−0.08	Good	2.375	12.977	22.83	0	4.728
15	REC‐1	MCL	−0.13	Good	3.63	13.392	12.589	0	6.329

* Rating of response based on T/C: good <0.3; moderate 0.3–0.6; weak >0.6.

CRC, colorectal cancer; DLBCL, diffuse large B‐cell lymphoma; GCB, germinal center B‐cell subtype; MCL, mantle cell lymphoma; NSCLC, non‐small cell lung cancer; SCLC, small cell lung cancer; TNBC, triple‐negative breast cancer.

Increased nuclear pCHK1 staining was correlated with higher elimusertib sensitivity, so we went on to validate the pCHK1 antibody used in this study for its specificity for its potential use in clinical diagnosis. MCF10A cells, a non‐transformed, human mammary epithelial cell line, were exposed to hydroxyurea, a cytotoxic agent that is known to cause stalled replication forks and activate CHK1 phosphorylation [22]. Accordingly, when compared with the untreated MCF10A cells, cells treated with hydroxyurea showed significantly increased pCHK1‐Ser345 levels by immunofluorescence (supplementary material, Figure S1A,B) and blotting (supplementary material, Figure S1C) assays. Furthermore, transient knockdown of CHK1 in these cells resulted in a complete loss of total and pCHK1 levels, thereby confirming that the antibody specifically binds to pCHK1.

### pCHK1 expression in biopsies from patients treated with the ATR inhibitor elimusertib

**Figure 4:**
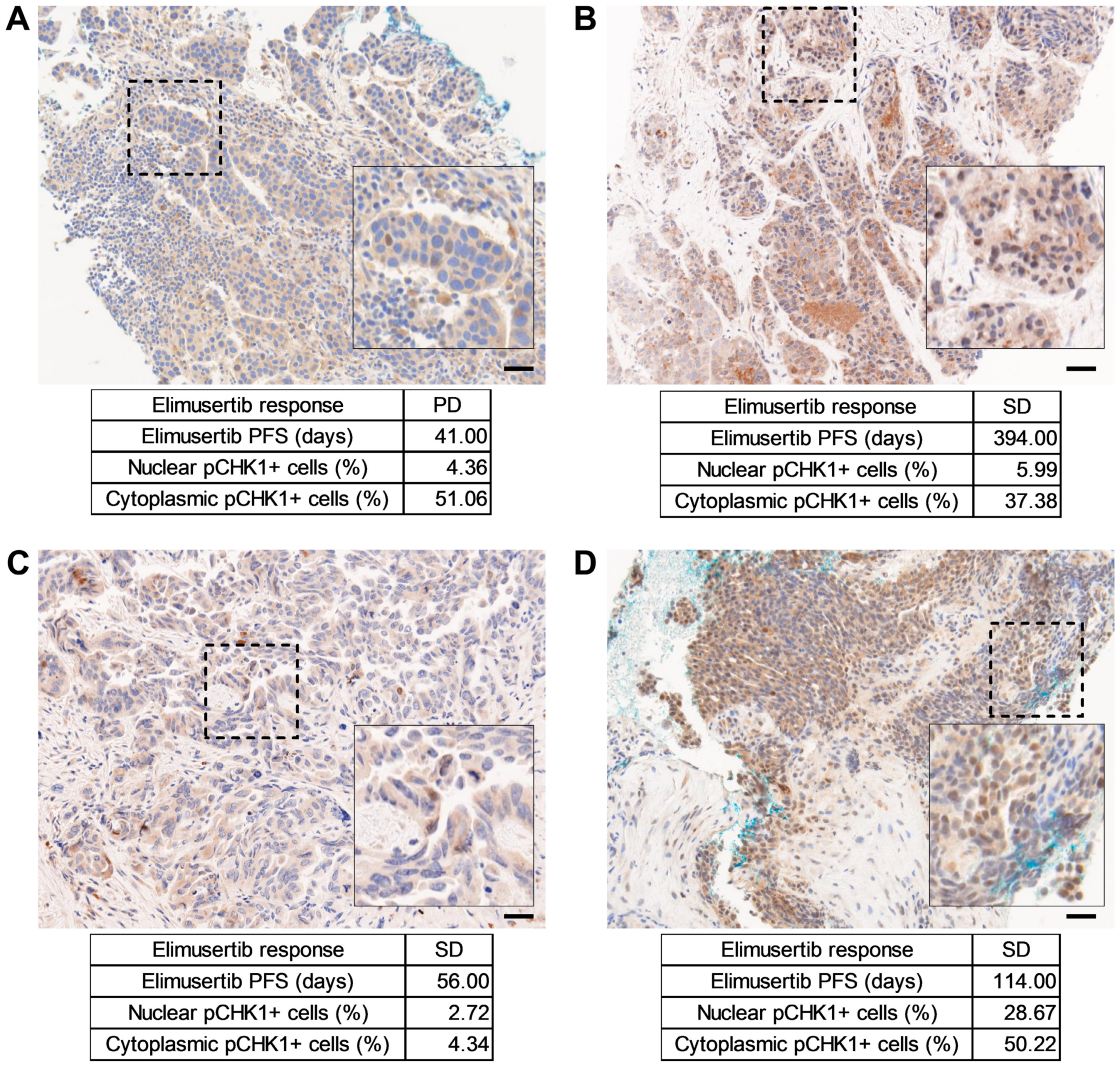
Nuclear pCHK1 staining is indicative of better survival in elimusertib‐treated patients. (A–D) Representative staining for pCHK1 on tumor biopsies obtained from patients immediately prior to commencing elimusertib therapy, showing (A) predominantly cytoplasmic versus (B–D) nuclear specific staining in cancer cells. Scale bars: 50 μm. The inset square area shown in each picture is enlarged from the region marked with a dashed frame. PD, progressive disease; SD, stable disease; PFS, progression‐free survival.

Archival pre‐treatment FFPE (formalin‐fixed, paraffin‐embedded) tumor tissue was retrospectively retrieved from four patients with high‐grade serous ovarian cancer who had been treated with elimusertib (NCT03188965) at the National University Health System, Singapore. The retrieved archival tumor tissue was from biopsies obtained immediately prior to elimusertib initiation. Amongst the four patients, review of medical records revealed that three patients had achieved stable disease (SD) as best response to elimusertib, while one patient had progressive disease (PD) as best treatment response by the RECIST 1.1 criteria. Using the automated Vectra 3 imaging system, we quantified nuclear versus cytoplasmic specific pCHK1 signals in these archival tissue samples. We observed that the patient with elimusertib‐resistant disease (Figure 4A) displayed the shortest progression‐free survival (PFS), 41 days, and relatively low levels of nuclear pCHK1 (4.36%). In contrast, the three patients with stable disease (Figure 4B–D) showed progression‐free survival times of 394, 56, and 114 days, with relatively higher nuclear pCHK1 localization (5.99%, 2.72%, and 28.67%, respectively). However, the levels of cytoplasmic pCHK1 staining remained fairly similar between patients with progressive disease and stable disease. In conclusion, in this small cohort of ovarian cancer patients, we observed a higher nuclear pCHK1 signal in patients who achieved greater clinical benefit from elimusertib treatment, which is consistent with earlier observations in our cancer cell line panels and cell line‐derived xenografts.

## Discussion

Previous work by Wengner *et al* [13] identified elimusertib, a highly selective inhibitor of ATR, as a promising candidate for the treatment of patients with tumors harboring DDR defects. Data from a phase I trial of elimusertib have demonstrated intriguing partial responses and prolonged SD in platinum‐ and PARPi‐resistant ovarian cancer patients with underlying DDR deficiency [14]. In the present study, we have extended our investigation to the identification of potential predictive biomarkers of elimusertib sensitivity. Our correlation of differential gene expression to elimusertib drug sensitivity in a spectrum of human cancer cell lines revealed upregulation of the DDR pathway genes (DNA repair/cell cycle checkpoint/apoptosis) in cell lines highly sensitive to elimusertib treatment. Furthermore, our panel of ovarian cancer cell lines that are sensitive to elimusertib showed higher levels of activated CHK1 (pCHK1). Of note, these ovarian cancer cell lines represent different molecular subtypes, epithelial–mesenchymal transition (EMT) status, histologies, and *TP53* mutation status (supplementary material, Table S4 [23]), indicating that our study sought to investigate the correlation of putative markers and elimusertib sensitivity in ovarian cancer in general, and not limited to a specific subgroup. These observations are consistent with previous studies in other tumor types investigating other cell‐cycle checkpoint inhibitors. For example, a cluster of diffuse large B‐cell lymphoma (DLBCL) cell lines and primary cells with constitutive expression of DDR pathway proteins (γH2AX and pCHK2) were sensitive to CHK1/CHK2 inhibitors [24], while primary glioblastoma cell lines with elevated levels of γH2AX were highly sensitive to the WEE1 inhibitor MK‐1775 [25]. Cancer cell lines that are sensitive to the CHK1 inhibitor MK‐8776 from multiple cancer entities have also shown rapid accumulation of CDK2‐activated DNA double‐stranded breaks [26]. Furthermore, human and hamster cell lines with high levels of DNA‐PKcs (DNA‐dependent protein kinase, catalytic subunit) were hypersensitive to the ATR inhibitor VE‐821 [27]. Therefore, these investigations highlight that cancer cell lines with elevated levels of DNA damage response activity are inherently susceptible to targeting through cell‐cycle checkpoint inhibitors.

Using an automated, multiplex immunofluorescence system, we showed that expression of pCHK1 was detected in the nucleus and/or in the cytoplasm of cell line‐derived xenograft samples and that the differential subcellular localization of CHK1 correlated variably with elimusertib sensitivity.

We observed that predominant cell line‐derived xenografts with a good response [treatment/control (T/C) ratio < 0.3] to elimusertib showed elevated nuclear pCHK1 staining levels, and therefore showed a marginal trend towards higher elimusertib sensitivity. The observed trend is further supported in our panel of ovarian cancer cell lines with higher sensitivity (lower IC_50_) to elimusertib, which showed significant enrichment of nuclear pCHK1 expression. These data suggest that xenografts and cell lines with higher levels of intrinsic replicative stress levels, as indicated by higher nuclear pCHK1 levels, would be more sensitive to elimusertib treatment. To our knowledge, these are the first pan‐cancer pre‐clinical data demonstrating that elevated nuclear pCHK1 levels predict sensitivity to an ATR inhibitor. Interestingly, in spite of the elevated expression in elimusertib‐sensitive cell lines, we noticed that nuclear specific upregulation of other candidate targets such as pATR and γH2AX in xenografts did not correlate with elimusertib sensitivity. The recruitment and stability of ATR to DNA lesions are mediated through its specific obligatory partner proteins. ATR forms a complex with ATR‐interacting protein (ATRIP) at single‐stranded DNA breaks [28, 29, 30], and associates with DNA‐PKcs at double‐stranded DNA lesions [31, 32]. Since none of these ATR binding partners were upregulated after elimusertib treatment in our panel of cell lines (supplementary material, Table S1), the accumulated nuclear pATR upregulation alone might not represent activated and stable ATR signaling. In addition, due to the functional redundancy of multiple kinases including ATR, ATM, and DNA‐PK involved in phosphorylating H2AX, ATR may not be regarded as a reliable predictor of elimusertib sensitivity [18, 33]. In contrast, CHK1 has been known to autonomously transduce the ATR‐mediated RSR pathway, and nuclear pCHK1 levels have been shown to predict early local recurrence and shorter survival in breast cancer patients [34, 35, 36]. Furthermore, amongst clinical tumor samples, patients with elevated nuclear pCHK1 expression at baseline achieved disease stabilization, and in some cases, durable clinical benefit, from elimusertib therapy.

Our multiplex immunofluorescence system involves automated and sequential labeling of antibodies using the Opal™ Tyramide Signal Amplification (TSA) system followed by the Vectra 3 spectral imaging system. Through covalent links, the TSA conjugated specific fluorophores emit stable signals when bound to targeted epitopes, facilitating multiplex staining methods and efficiently circumventing any issues involving antibody species cross‐reactivity [37]. Also, the automated Vectra 3 imaging system has been shown to be an efficient multiplex imaging tool even in challenging scenarios such as the concurrent scoring of markers identifying tumor and immune cell populations of heterogeneous lymphoma [38]. Furthermore, experimental models that are currently used for assessing cellular replicative stress levels, such as DNA fiber assay, and nascent iPOND techniques remain inapplicable for clinical samples without intermediate study models like primary cancer cell lines and tumor‐derived organoids. Therefore, our multiplex immunofluorescence system‐mediated assessment of DDR‐ or RSR‐associated proteins on xenograft and clinical samples renders a pragmatic solution. Our study is limited by the lack of a larger cohort of pre‐treatment patient samples to clinically validate the predictive value of nuclear CHK1 expression. Nonetheless, our findings suggest that further evaluation of this marker is warranted in completed and ongoing studies of ATR inhibitors being tested in clinical trials. It is envisaged that future research focused on identifying additional DDR‐ or RSR‐associated proteins that are predictive of sensitivity to RSR targeted drugs could pave the way to enhancing patient selection for these inhibitors in the context of targeted cancer therapy.

## Author contributions statement

DSPT conceived the study. VS, TZT, DGZL, YP, AMW, NYLN and ADJ contributed to the methodology. All the authors contributed to investigation and visualization. DSPT was responsible for acquisition of funding. ADJ and DSPT were responsible for project administration and supervision. VS, TZT, NYLN and DSPT wrote the original draft, and all the authors contributed to revisions and editing.

## Supporting information


**Supplementary materials** **and methods**
**Figure S1.** Validation of the pCHK1‐Ser345 antibody
**Table S1.** Cell lines and their respective culture media
**Table S2.** Markers and fluorophores used for multiplex immunofluorescence staining
**Table S3.** Correlation analysis of significantly expressed genes in the PanCancer pathway panel
**Table S4.** Molecular and histological background of the 15 ovarian cancer cell lines used in the study

## Data Availability

The NanoString PanCancer pathway panel profiling from 24 cancer cell lines used in this study has been deposited in NCBI's Gene Expression Omnibus (GEO) repository and is accessible through GEO Series accession number GSE217579 (https://www.ncbi.nlm.nih.gov/geo/query/acc.cgi?acc=GSE217579).
