# Supplementary material for: Nuclear pCHK1 as a potential biomarker of increased sensitivity to ATR inhibition
Source: J Pathol. 2022 Dec 8;259(2):194–204. doi: 10.1002/path.6032 (PMC10107453; doi:10.1002/path.6032)
Supplement: Supplementary file 1 — Supplementary materials and methods Figure S1. Validation of the pCHK1‐Ser345 antibody Table S1. Cell lines and their respective culture media Table S2. Markers and fluorophores used for multiplex immunofluorescence staining Table S3. Correlation analysis of significantly expressed genes in the PanCancer pathway panel Table S4. Molecular and histological background of the 15 ovarian cancer cell lines used in the study [file PATH-259-194-s001.docx]

**Nuclear pCHK1 as a potential biomarker of increased sensitivity to ATR inhibition**

V Sundararajan *et al. J Pathol* <https://doi.org/10.1002/path.6032>

**Supplementary materials and methods**

**Figure S1**

**Tables S1–S4**

**SUPPLEMENTARY MATERIALS AND METHODS**

Reference numbers refer to the main text list

*Single marker immunohistochemistry*

Single immunohistochemical staining for pCHK1 was performed through the Leica BondMax system (Serial No. M211523). A Leica Biosystems Bond Polymer Refine Detection Kit (Leica Biosystems, Newcastle Upon Tyne, UK; Cat # DS9800) was used for this study. In brief, the slides were baked and dewaxed, followed by heat-induced epitope retrieval (HIER) using HIER 2 solution (Leica Biosystems Bond Epitope Retrieval 2-1L; Cat # AR9640) at 100 °C for 20 min. The slides were then peroxidase-blocked for 10 min, followed by incubation with primary antibodies in antibody diluent (Antibody Diluent with Background-Reducing Components; Agilent Dako, Santa Clara, USA; Cat # DKO.S302283). Subsequently, the polymeric HRP-conjugated secondary antibody, DAB, and hematoxylin reagents (from the Bond Polymer Refine Kit, Leica Biosystems) were dispensed onto the slides sequentially. Slides were rinsed with 1× washing buffer (Leica Biosystems BondTM Wash Solution 10X Concentrate, Cat # AR9590) after each step. After staining, the slides were mounted using Leica CV Ultra mounting medium (Leica Biosystems; Cat # 14070936261). The Vectra 3 system (PerkinElmer Inc., Waltham, MA, USA) was used to image the slides after IHC staining. The slides were reviewed together with a control tonsil tissue slide. Each image was manually reviewed to assess the presence of both DAB and hematoxylin staining. Signals were presented as actual color (as for Figure 4).

*Multispectral imaging and signal quantification*

The Vectra 3 system (PerkinElmer Inc.) was used to image the TMA slide after multiplex immunofluorescence staining. The TMA slide was reviewed together with a control slide and an auto-fluorescence control slide. The optimal exposure times for each fluorescence channel were set up until there were no overexposed regions in the live camera image and reference images were acquired. All slides were scanned in Vectra 3 TMA scanning mode with all cores on the slides exposed. Each image was manually reviewed to assess the presence of each fluorophore and to ensure no mixing between the fluorophores. Each image captured was converted to pseudo-color, mimicking a DAB-based IHC stain for ease of visualization. Using Inform 2.4.2, an optimal algorithm was applied for cell segmentation, whereas tissue segmentation was performed by manually removing any region that would cause errors for data analysis, such as folded areas and muscle tissues. Thresholds for each marker were set for each sample core (Vectra ID). Accordingly, the mean intensities of the stained markers – geminin (nuclear), pATR (nuclear), pCHK1 (nuclear and cytoplasmic), and γH2AX (nuclear) – for each of the xenograft samples were attained.

Image signals were unmixed and scaled relative to all sample images in the batch using Inform 2.4.2 software (PerkinElmer Inc.). Signals were presented as pseudo-color (as for Figure 3A and supplementary material, Figure S1A) and are indicated in the figure legends where applicable.

*siRNA treatment and western blotting*

To validate the specificity of the pCHK1 antibody, MCF10A cells were treated with 30 nm of control siRNA – ON-TARGET plus Non-targeting Control Pool (Horizon Discovery, Cambridge, UK; Cat # D-001810-10-05). CHK1 siRNAs [CHK1 si-1 (5'-GCAACAGUAUUUCGGUAUATT-3'), CHK1 si-2 (5'-GGACUUCUCUCCAGUAAACTT-3')] were self-designed and synthesized by SABio, Singapore using Lipofectamine RNAiMAX transfection reagent (Thermo Fisher Scientific, Waltham, MA, USA; Cat # 13778150) for 24 h. Subsequently, cells were left untreated or treated with 1 mm hydroxyurea (Sigma, St Louis, MO, USA; Cat # H8627) for 24 h. After treatment, cell pellets were collected and embedded in paraffin blocks and processed for staining. For western blotting, whole cell lysates were prepared using RIPA buffer and resolved using standard SDS-PAGE electrophoresis. Cytoplasmic and nuclear extracts were prepared using a nuclear extract kit (Active Motif, Carlsbad, CA, USA; Cat # 40010, # 37512, # 37517) kindly gifted by Dr Azhar Ali, CSI Singapore. Immunoblots were incubated with primary antibodies [anti-P-ATR (Ser428; Abcam, Cambridge, UK; Cat # ab178407; 1:1,000); anti-P-CHK1 (Ser345; Cell Signaling Technology, Danvers, MA, USA; Cat # 2348; 1:500); anti-gamma H2AX (Ser139, Abcam, Cat # ab11174; 1:500); anti-total ATR (Cell Signaling Technology, Cat #2790; 1:1,000); anti-total CHK1 (Cell Signaling Technology, Cat #2360; 1:1,000); anti-total H2AX (Bethyl Laboratories, Montgomery, TX, USA; Cat # A303-837A; 1:1,000); anti-Histone-H3 (Abcam, Cat # ab24834; 1:1,000); anti-GAPDH (Cell Signaling Technology, Cat # 2118; 1:5,000)]. Infrared dye-conjugated secondary antibodies from LI-COR Biosciences, Lincoln, NE, USA [IRDye 800-CW goat anti-mouse or anti-rabbit (# 926-32210; 1:10,000 and # 926-32211; 1:10,000) and IRDye 680LT goat anti-mouse or anti-rabbit (# 926-68020; 1:10,000 and # 926-68021; 1:10,000)] were used for quantitative detection. Blots were scanned using the Odyssey infrared imaging system (LI-COR), and the resulting images were transferred to greyscale. Protein band densities were analyzed using ImageJ software [39] and quantified as described using Davarinejad’s protocol [40].

**Figure S1**


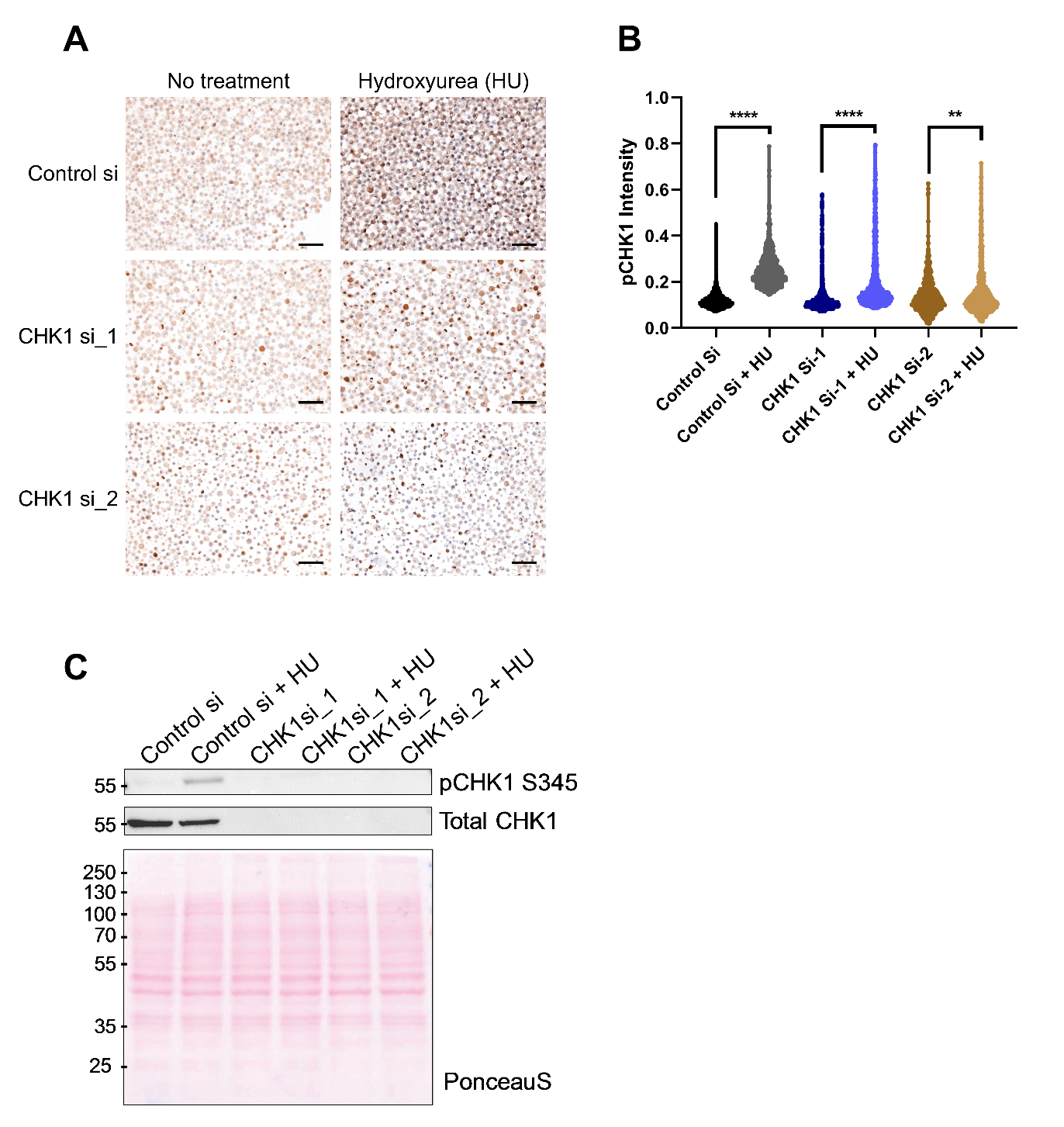


**Figure S1. Validation of the pCHK1-Ser345 antibody.** (A) Images of control and CHK1 knockdown MCF10A cell blocks with/without treatment using hydroxyurea (HU) and stained for pCHK1-Ser345. Scale bar: 40 µm. Each image was obtained through multiplex immunofluorescence staining and is presented as pseudo-color, mimicking a DAB-based IHC stain for ease of visualization. (B) Violin plot showing quantification of signal intensities from at least 800 individual cells. One-way ANOVA and Tukey’s multiple comparisons test were used to compute statistical significance. ***p* < 0.01; *****p* < 0.0001. (C) Immunoblots showing expression of indicated proteins in control and CHK1 knockdown cells with/without treatment using HU. Ponceau S staining was used as a loading control.

**Supplementary Tables S1–S4**

| **Table S1. Cell lines and their respective culture media.** | | | | | |
| --- | --- | --- | --- | --- | --- |
| S. No. | Tissue of origin | Cell line / model | adherent (adh) / suspension (susp) | Medium | FCS (%) |
| 1 | ABC-DLBCL | HBL-1 | susp | RPMI 1640 | 10 |
| 2 | ABC-DLBCL | TMD-8 | susp | RPMI 1640 | 20 |
| 3 | Breast | MDA-MB-436 | adh | DMEM/Ham´s F12 | 10 |
| 4 | Cervix | HeLa-MaTu | adh | DMEM/Ham´s F12 | 10 |
| 5 | Cervix | HeLa-MaTu-ADR | adh | DMEM/Ham´s F12 + FCS + doxorubicin (0.1 µg/ml) | 10 |
| 6 | CRC | HCT-116 | adh | DMEM/Ham´s F12 | 10 |
| 7 | CRC | HT-29 | adh | DMEM/Ham´s F12 | 10 |
| 8 | CRC | Lovo | adh | RPMI 1640 | 10 |
| 9 | GCB-DLBCL | SUDHL-6 | susp | RPMI 1640 | 10 |
| 10 | MCL | GRANTA-519 | susp | DMEM | 10 |
| 11 | MCL | Jeko-1 | susp | RPMI 1640 | 10 |
| 12 | MCL | JVM-2 | susp | RPMI 1640 | 10 |
| 13 | MCL | REC-1 | susp | RPMI 1640 | 10 |
| 14 | Melanoma | HT-144 | adh | McCoy's 5A + FCS + l-alanyl-l-glutamine (2 mm) | 10 |
| 15 | Multiple myeloma | OPM-2 | susp | RPMI 1640 | 10 |
| 16 | NSCLC | A549 | adh | DMEM/Ham´s F12 | 10 |
| 17 | Ovarian | A2780 | adh | RPMI 1640 | 10 |
| 18 | Ovarian | DOV13 | adh | DMEM | 10 |
| 19 | Ovarian | HEY | adh | RPMI 1640 | 10 |
| 20 | Ovarian | HEYA8 | adh | RPMI 1640 | 10 |
| 21 | Ovarian | HEYC2 | adh | RPMI 1640 | 10 |
| 22 | Ovarian | IGROV-1 | adh | RPMI 1640 | 10 |
| 23 | Ovarian | JHOS4 | adh | DMEM/F12 (1:1), 0.1 mm NEAA | 10 |
| 24 | Ovarian | OV17R | adh | DMEM/F12 (1:1), 0.4 μg/ml hydrocortisone, 10 μg/ml insulin | 5 |
| 25 | Ovarian | OVCA429 | adh | DMEM | 10 |
| 26 | Ovarian | OVCA433 | adh | DMEM | 10 |
| 27 | Ovarian | PEO1 | adh | RPMI 1640 | 10 |
| 28 | Ovarian | PEO4 | adh | RPMI 1640 | 10 |
| 29 | Ovarian | SKOV3 | adh | RPMI 1640 | 10 |
| 30 | Ovarian | TYKNU | adh | DMEM | 10 |
| 31 | Ovarian | UWB1.289 | adh | RPMI 1640/ MEGM (1:1) | 3 |
| 32 | Pancreas | MiaPaCa2 | adh | DMEM/Ham’s F12 + FCS + horse serum (2.5%) | 10 |
| 33 | Prostate | 22Rv1 | adh | RPMI 1640 | 10 |
| 34 | Prostate | LapC4 (internal) | adh | RPMI 1640 + FCS + R1881 (1 nm) | 10 |
| 35 | Prostate | PC3 | adh | DMEM/Ham´s F12 | 10 |
| 36 | SCLC | NCI-H82 | susp | RPMI 1640 | 10 |
| 37 | TNBC | MFM223 | adh | MEM Earle’s + FCS + l-alanyl-l-glutamine (2 mm) | 10 |

**Table S2. Markers and fluorophores used for multiplex immunofluorescence staining**

| **Markers** | **Opal fluorophores** | **Excitation wavelength** | **Emission wavelength** |
| --- | --- | --- | --- |
| Geminin | Opal 520 | 494 nm | 525 nm |
| pATR | Opal 540 | 523 nm | 536 nm |
| pCHK1 | Opal 570 | 550 nm | 570 nm |
| γH2AX | Opal 620 | 588 nm | 616 nm |
| PD-L1 | Opal 690 | 676 nm | 694 nm |

**Table S3. Correlation analysis of significantly expressed genes in the PanCancer pathway panel.**

| S. No. | Index | Param/  gene | Pathway | Mean expression | Correlation_versus_ATRi IC50 (nM)_Pearson.*Rho* | Correlation_versus_ATRi IC50 (nM)_Pearson.*p* value |
| --- | --- | --- | --- | --- | --- | --- |
| 1 | 210 | FAS | MAPK;CC+Apop | 6.1030 | −0.6640 | 0.0004 |
| 2 | 293 | HDAC4 | ChromMod | 6.5506 | −0.6470 | 0.0006 |
| 3 | 286 | H2AFX | DNARepair | 10.0848 | −0.6450 | 0.0007 |
| 4 | 92 | CCND1 | Wnt;JAK-STAT;PI3K;CC+Apop | 8.9703 | −0.6351 | 0.0009 |
| 5 | 649 | SOS2 | MAPK;JAK-STAT;PI3K;RAS | 6.6938 | −0.6286 | 0.0010 |
| 6 | 616 | RUNX1T1 | TXmisReg | 1.2008 | 0.6021 | 0.0019 |
| 7 | 312 | HRAS | Driver Gene;MAPK;PI3K;RAS | 8.7959 | −0.5979 | 0.0020 |
| 8 | 706 | UBB | DNARepair | 13.3384 | −0.5714 | 0.0035 |
| 9 | 623 | SFRP2 | Wnt | 0.5453 | 0.5630 | 0.0042 |
| 10 | 493 | NTRK1 | TXmisReg;MAPK;CC+Apop | 0.3824 | 0.5630 | 0.0042 |
| 11 | 406 | LEFTY2 | TGF-B | 0.4039 | 0.5630 | 0.0042 |
| 12 | 338 | IL12RB2 | JAK-STAT | 0.1207 | 0.5630 | 0.0042 |
| 13 | 352 | IL23A | JAK-STAT | 3.0378 | −0.5574 | 0.0047 |
| 14 | 93 | CCND2 | Wnt;TXmisReg;JAK-STAT;PI3K;CC+Apop | 4.8488 | 0.5516 | 0.0052 |
| 15 | 270 | GNA11 | Driver Gene | 9.0545 | −0.5426 | 0.0061 |
| 16 | 193 | EPOR | JAK-STAT;PI3K | 3.4499 | −0.5359 | 0.0069 |
| 17 | 515 | PHF6 | Driver Gene | 10.3061 | 0.5353 | 0.0070 |
| 18 | 425 | MAP3K5 | MAPK | 6.3952 | −0.5320 | 0.0075 |
| 19 | 472 | NFE2L2 | Driver Gene | 10.5970 | −0.5271 | 0.0081 |
| 20 | 545 | POLD1 | DNARepair | 6.8055 | 0.5262 | 0.0083 |
| 21 | 199 | ETV4 | TXmisReg | 6.8427 | −0.5119 | 0.0105 |
| 22 | 67 | CACNA2D4 | MAPK | 0.3460 | 0.5099 | 0.0109 |
| 23 | 34 | BAIAP3 | TXmisReg | 2.6947 | −0.4982 | 0.0132 |
| 24 | 617 | RXRG | TXmisReg | 1.5289 | 0.4944 | 0.0141 |
| 25 | 111 | CDK4 | PI3K;CC+Apop | 10.9722 | −0.4935 | 0.0143 |
| 26 | 648 | SOS1 | MAPK;JAK-STAT;PI3K;RAS | 8.2512 | 0.4935 | 0.0143 |
| 27 | 187 | EGFR | Driver Gene;MAPK;PI3K;RAS | 6.9597 | −0.4904 | 0.0150 |
| 28 | 47 | BMP4 | HH;TGF-B | 4.0726 | −0.4902 | 0.0150 |
| 29 | 285 | GZMB | TXmisReg | 1.2998 | 0.4896 | 0.0152 |
| 30 | 178 | DUSP6 | TXmisReg;MAPK | 6.9219 | −0.4750 | 0.0190 |
| 31 | 396 | LAMA1 | PI3K | 1.6337 | 0.4720 | 0.0199 |
| 32 | 460 | MTOR | PI3K | 8.7511 | 0.4716 | 0.0200 |
| 33 | 320 | ID2 | TXmisReg;TGF-B | 9.6694 | −0.4645 | 0.0222 |
| 34 | 246 | FOS | MAPK | 7.3115 | −0.4556 | 0.0252 |
| 35 | 414 | LTBP1 | TGF-B | 7.1535 | −0.4513 | 0.0268 |
| 36 | 110 | CDK2 | PI3K;CC+Apop | 8.5983 | −0.4472 | 0.0284 |
| 37 | 123 | CHEK1 | CC+Apop | 9.9004 | −0.4394 | 0.0317 |
| 38 | 49 | BMP6 | TGF-B | 3.6642 | −0.4373 | 0.0326 |
| 39 | 135 | COL2A1 | PI3K | 6.3728 | 0.4361 | 0.0332 |
| 40 | 216 | FGF11 | MAPK;PI3K;RAS | 3.8908 | 0.4350 | 0.0337 |
| 41 | 470 | NF2 | Driver Gene | 9.9596 | −0.4329 | 0.0346 |
| 42 | 9 | ACVR2A | TGF-B | 6.2252 | −0.4313 | 0.0353 |
| 43 | 346 | IL1R2 | TXmisReg;MAPK | 1.7704 | −0.4310 | 0.0355 |
| 44 | 54 | BRAF | Driver Gene;MAPK | 8.8850 | 0.4290 | 0.0364 |
| 45 | 399 | LAMB3 | PI3K | 4.8033 | −0.4212 | 0.0404 |
| 46 | 653 | SP1 | TXmisReg;TGF-B | 7.9761 | −0.4209 | 0.0406 |
| 47 | 378 | ITGB6 | PI3K | 2.5152 | −0.4192 | 0.0415 |
| 48 | 600 | RB1 | Driver Gene;CC+Apop | 9.9238 | −0.4187 | 0.0417 |
| 49 | 679 | THBS1 | TGF-B;PI3K | 6.1162 | −0.4176 | 0.0423 |
| 50 | 497 | OSM | JAK-STAT;PI3K | 1.6896 | −0.4132 | 0.0448 |
| 51 | 719 | WNT16 | Wnt;HH;TXmisReg | 1.8479 | −0.4127 | 0.0451 |
| 52 | 516 | PIK3CA | Driver Gene;JAK-STAT;PI3K;RAS;CC+Apop | 9.2815 | −0.4115 | 0.0457 |
| 53 | 633 | SKP1 | Wnt;TGF-B;CC+Apop | 13.3428 | −0.4112 | 0.0459 |
| 54 | 526 | PKMYT1 | CC+Apop | 7.8263 | −0.4106 | 0.0463 |
| 55 | 213 | FEN1 | DNARepair | 10.6870 | −0.4102 | 0.0465 |
| 56 | 159 | DAXX | Driver Gene;MAPK | 9.9051 | 0.4071 | 0.0483 |
| 57 | 31 | AXIN2 | Wnt | 3.6468 | −0.4050 | 0.0496 |

**Table S4. Molecular and histological background of the 15 ovarian cancer cell lines used in the study.**

| **SN** | **Name** | **Subtype** | **EMT phenotype** | **EMT score** | **Histology** | ***TP53* status** |
| --- | --- | --- | --- | --- | --- | --- |
| 1 | UWB1.289 | EpiA | IE | −0.24 | Papillary serous | R209del2b |
| 2 | TykNu | Mes | M | 0.773333 | Undifferentiated | R175H |
| 3 | JHOS4 | StemA | IE | −0.28 | Serous cystadenocarcinoma | – |
| 4 | OV17R | StemA | IE | 0.513333 | Adenocarcinoma | V216M |
| 5 | PEO1 | EpiA | E | −0.65333 | Poorly differentiated serous adenocarcinoma | G244D |
| 6 | A2780 | StemA | M | 0.973333 | Undifferentiated | WT |
| 7 | DOV13 | Mes | IM | 0.186667 | Adenocarcinoma | WT |
| 8 | SKOV 3 | Mes | IM | 0.38 | Serous | S90del1b (base 267 del C) |
| 9 | HeyA8 | Mes | M | 0.44 | Serous | WT |
| 10 | IGROV1 | StemA | IE | 0.173333 | Endometrioid | Y126Y/C |
| 11 | OVCA433 | EpiA | IE | −0.68667 | Papillary serous cystadenocarcinoma | WT |
| 12 | OVCA429 | EpiA | IE | −0.58 | Serous | WT |
| 13 | PEO4 | EpiA | IE | 0.6 | Poorly differentiated serous adenocarcinoma | G244D |
| 14 | HeyC2 | Mes | IM | 0.506667 | Serous | WT |
| 15 | Hey | Mes | IM | 0.333333 | Serous | WT |

SN, sample number.
